# Application of Atlas-based global shape and local contraction analysis to single-ventricle congenital heart disease

**DOI:** 10.1186/1532-429X-18-S1-P154

**Published:** 2016-01-27

**Authors:** Avan Suinesiaputra, Genevieve E Farrar, Kathleen Gilbert, Sanjeet Hegde, Andrew D McCulloch, Jeff Omens, James C Perry, Alistair Young

**Affiliations:** 1 Department of Anatomy with Radiology, University of Auckland, Auckland, New Zealand; 2 Department of Bioengineering, Universeity of California San Diego, San Diego, CA USA; 3 Division of Cardiology, Rady Children's Hospital, San Diego, CA USA

## Background

Improvements in palliative surgery for infants born with single ventricle heart defects have increased their survival rate into adulthood, but the risk of developing heart failure remains high. The ventricles remodel to geometric extremes, making statistical comparison with the normal population difficult beyond the assessment of mass and volumes. We present a new method for evaluating cardiac geometry and systolic contraction variations in patients with single ventricle physiology with respect to the normal range of end-diastolic (ED) and end-systolic (ES) left ventricle (LV) shapes seen in the asymptomatic population.

## Methods

Cardiac MRI of four single-ventricle patients with tricuspid atresia and > 10 years post-Fontan surgery were analyzed using custom software (CIM, Auckland, New Zealand). EDV (ml) = {183.5, 122.9, 123.6, 77.7}, ESV (ml) = {97.7, 60.9, 72.7, 35.3}, EF (%) = {46.8, 50.5, 41.2, 54.6}, and LVM (g) = {143.4, 116.8, 134.9, 63.3}. Each patient's LV shape was projected onto Principal Component Analysis (PCA) models previously derived from 1,991 asymptomatic volunteers [1], resulting as patient's principal scores for each PCA component. For local contraction analysis, thin-plate spline registration aligned LV shapes at ED onto the mean ED shape of the asymptomatic group. The corresponding ES shape was then deformed following the same ED shape alignment causing each patient LV to start the contraction from the same shape. ES points were compared statistically using the Mahalanobis distance.

## Results

**Figure 1:**
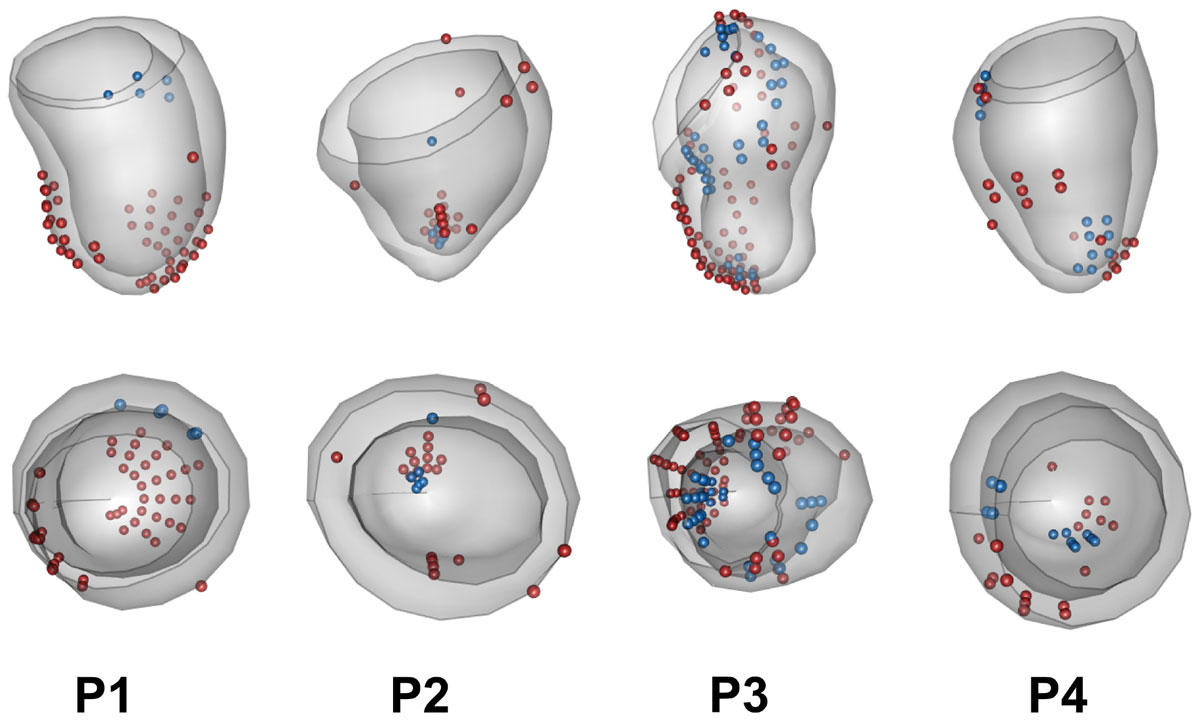
**LV shapes at ED from four single-ventricle patients**. Spheres show ES points that lie outside the asymptomatic distributions (blue points are on endosurface, red points are on episurface). The bottom row shows view from the base with septum on the left side.

Geometric LV shape extremes were identified at ED for P2 and P3 (Figure 1), even though LV volumes and masses were within normal ranges. Standardised principal scores of the four patients with respect to the asymptomatic group from the first five components are shown in Table 1. Individually, P4 had a small ventricular size, while P3 had a large inclination of the basal region affecting the LV outflow tract variation. P2 had a shorter height, a lower basal plane, and a thinner wall. For regional contraction, Figure 1 shows ES points after the ED alignment that lie outside the asymptomatic distributions. Each patient showed different patterns of local contraction abnormalities. P1 had abnormal lateral and septal epicardial wall contraction, while P3 shows abnormal contraction in multiple regions.

**Table 1:** Principal scores of four single-ventricle patients (P1-P4) after projecting onto PCA models of 1,991 asymptomatic subjects.

PC	Geometry variation	Pct	P1	P2	P3	P4
PCA-ED						
1	Size	44.5%	-0.9	1.6	-0.7	**2.2**
2	Base Plane	10.6%	0.6	**3.1**	1.6	-0.2
3	Basal Slice	9.2%	1.3	1.4	**-6.6**	0.5
4	Longitudinal (height)	6.8%	-1.5	**-3.1**	-1.6	0.1
5	Apical Slice	5.0%	-0.4	-2.0	-1.5	-0.4
**PCA-ES**						
1	Size	43.1%	-1.3	0.4	-0.8	1.5
2	LV Outflow Tract	11.0%	-1.2	-1.8	**3.3**	**-2.7**
3	Basal Thickness	7.2%	-0.2	**-2.7**	-0.8	0.8
4	Mid-lateral Wall	5.0%	**-3.4**	-1.3	**2.9**	**-2.3**
5	Wall Thickness	4.3%	1.3	1.2	**4.2**	1.4

Only the first five principal components (PC) are shown. Pct = percentage of variance explained. Values are the standardised principal scores; highlighted with bold face are values outside ± 2 standard deviation.

## Conclusions

Atlas-based statistical shape analysis allows for identification of abnormal shape characteristics beyond mass and volume, as well as regional systolic contraction patterns. This may provide valuable new measures of disease progression and remodeling into heart failure relative to population norms.
